# The Chemical Exposome of Human Aging

**DOI:** 10.3389/fgene.2020.574936

**Published:** 2020-11-23

**Authors:** Biswapriya B. Misra

**Affiliations:** Independent Researcher, Guntur, India

**Keywords:** exposome, metabolite, aging, mass spectrometry, informatics, pollution, model, telomere

## Abstract

Aging is an inevitable biological phenomenon displayed by single cells and organs to entire organismal systems. Aging as a biological process is characterized as a progressive decline in intrinsic biological function. Understanding the causative mechanisms of aging has always captured the imagination of researchers since time immemorial. Although both biological and chronological aging are well defined and studied in terms of genetic, epigenetic, and lifestyle predispositions, the hallmarks of aging in terms of small molecules (i.e., endogenous metabolites to chemical exposures) are limited to obscure. On top of the endogenous metabolites leading to the onset and progression of healthy aging, human beings are constantly exposed to a natural and anthropogenic “chemical” environment round the clock, from conception till death, affecting one’s physiology, health and well-being, and disease predisposition. The research community has started gaining sizeable insights into deciphering the aging factors such as immunosenescence, nutrition, frailty, inflamm-aging, and diseases till date, without much input from their interaction with exogenous chemical exposures. The “exposome” around us, mostly, accelerates the process of aging by affecting the internal biological pathways and signaling mechanisms that result in the deterioration of human health. However, the entirety of exposome on human aging is far from established. This review intends to catalog the known and established associations of the exposome from past studies focusing on aging in humans and other model organisms. Further discussed are the current technologies and informatics tools that enable the study of aging exposotypes, and thus, provide a window of opportunities and challenges to study the “aging exposome” in granular details.

## Introduction

The human exposome consists of the entirety of life-course environmental exposures and the lifestyle factors, a variable and dynamic entity which evolves throughout the lifetime of an individual and consists of every exposure to which an individual is subjected, from conception till death (Wild, 2005). Thus, the phenotype (P) is a sum of genotype (G) and its interaction with the environment (E), i.e., P = G + E. In the context of human health and disease the contribution of genotype for complex traits are low and variable, thus providing necessary impetus to research on the role of exposures on human health. These exposures around human civilization are not only natural but also engineered/anthropogenic factors that affect us every moment, either physically (i.e., noise) or chemically (i.e., pollutants). Xenobiotics such as pharmaceuticals and personal care products (PPCPs), pesticides and insecticides, plant-, bacteria- and food- derived compounds, food additives, surfactants, solvents, synthetic and industrial chemicals, and microbial compounds constitute a huge chunk of human chemical exposome (Misra, 2019). In a recent review (Vermeulen et al., 2020), the major categories of “chemical exposome” were identified as diet, drug use, smoking, alcohol use under lifestyle factors. Though most of the chemical exposures are unintentional, intentional exposures can happen too, whether be it for enjoyment (e.g., addictions such as smoking and alcoholism) or as side effects of a therapeutic treatment (e.g., chemotherapy, antibiotics etc.) (Sorrentino et al., 2014), the chemical exposures affect human life incessantly. Further, incidental exposures [e.g., polychlorinated biphenyls (PCBs), lead (Pb), nitrosamines, and pesticides] are also associated with chronic human diseases (Sorrentino et al., 2014). In a recent review, chemical exposures such as those from medication and environmental agents have been shown to be associated with metabolic/lifestyle diseases such as type 2 diabetes mellitus (T2DM) (Misra and Misra, 2020). In this review, the authors summarized that the disease predisposition was associated with exposure (levels) of POPs, phthalates, antibiotics, drugs, air pollution, pesticides, and heavy metals. An accumulating body of knowledge of the redox proteome and the redox interface of the genome and exposome further indicates that aging is a cumulative failure of the adaptive structures supporting genome–exposome interaction (Jones, 2015), given the continual interaction of exposome with the endogenous proteome, epigenome, genome (Go and Jones, 2014), and metabolome of human beings.

Aging in humans is defined as an inevitable progressive decline in physiology that leads to an increased risk of debility, disease, and eventual death. This physiological (and metabolic) decline is identified as the primary risk factor for important human diseases (López-Otín et al., 2013). The primary hallmarks (causes of damage) are identified as an unstable genome, shortening of telomeres, loss of proteostasis, and epigenetic modifications (López-Otín et al., 2013). The authors suggested other hallmarks as dysregulated nutrient sensing, functional imbalance in mitochondria, senescence, stem cell exhaustion, and dysregulated intercellular communication. Measurable biomarkers of aging common to mice and humans include senescence-associated beta-galactosidase (SA-β-gal) staining, leukocyte telomere length, interleukin 6 (IL-6), other SA-cytokines, tumor suppressor protein *p16INK4a* (an inhibitor of CDK4) mRNA in T lymphocytes, and DNA methylation (Sorrentino et al., 2014). Exposures to certain toxicants (named as “gerontogens”) enhance physiological aging (Sorrentino et al., 2014) as well. Exposure to unknown gerontogens explains much of the non-genetic variation in the rates of physiological aging processes (Sorrentino et al., 2014). A gradual slowing down of metabolism with age is also documented. A very recent review on the “aging metabolome” enumerated the set of biomarkers and hub metabolites of aging, indicating critical roles for nicotinamide adenine dinucleotide (NAD^+^), reduced nicotinamide dinucleotide phosphate (NADPH), α-ketoglutarate, and β−hydroxybutyrate with a central role of the TCA cycle in signaling and metabolic dysregulation associated with aging (Sharma and Ramanathan, 2020). As very recently commented by Di Ciaula and Portincasa, “successful aging could begin during in utero life” (Di Ciaula and Portincasa, 2020). However, there are currently no available literature where aging has been looked through the lenses of chemical exposures at an omics scale, i.e., an exposomal basis of aging. The connection between our chemical environment and its role in human aging is far from established, and we are just beginning to explore.

For the purpose of this review article, the term “environment” refers only to specify the “macroenvironment” (i.e., the habitat and dwelling space) and not the “microenvironment” such as those inside cells, organelles, and tissues etc. as noted elsewhere (Misra, 2019). Also, the term “exposome” is used in this review to exclusively include exogenous chemicals, and not the physical exposures such as noise, radiation, water, soil, education, societal, spiritual, occupational, and those different from the internal exposome (i.e., metabolism, physical activity, genetics, endocrine factors, inflammation, chronic diseases, stress etc.) (Misra, 2019).

**FIGURE 1:**
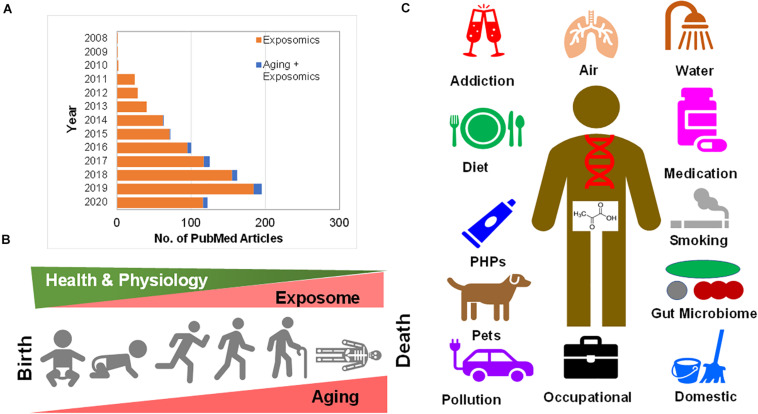
**(A)** Growth in research publications at PubMed pertaining to “exposomics” and “exposomics and aging” research. **(B)** Human aging from child birth (neonatal) through adolescence and the ultimate of life, death are riddled with exposure to chemicals surrounding us. As with the incremental aging one experiences physiological decline, possibly the cumulative exposome increases inside human body. **(C)** Human chemical exposure ranges from addiction (alcohol, drugs, cocaine, and smoking), to air pollution (PM, gases, industrial vapors), water (pollutions, pesticides, and PFAS), drugs (pharmaceuticals, and medications), the resident internal and external gut microbiome, domestic waste and indoor pollution (dust, cooking, and gases), occupational exposures (hospital disinfectants to asbestosis), pollution (fossil fuel burning to plastic), pets (carrying environmental pollution to microbiome), personal hygiene products (PHPs) (perfumes to soaps) to diet (and nutrition).

Available literature was screened from 1980 to 2020 (till date of communication) at PubMed (Figure 1A), Google Scholar, Scopus and bioRxiv literature databases manually for information content with various key words and their combinations such as: “exposome,” “exposomics,” “chemical,” exposures with “aging” and “age.” The output was manually curated to include accessible scholarly articles that were in English. The limited resulting literature combining aging and exposure studies encompass biological to epidemiological studies that span *in vitro* cell lines, animal models such as mice, rat, and *Caenorhabditis elegans* based studies, to those investigated human cohorts, i.e., epidemiological studies. Also, to avoid the complexity of diet/nutrition (i.e., duration, type, diversity etc.) as an exposure which may include hundreds of epidemiological and intervention studies, I have excluded these studies, as the diets themselves remain chemically complex and uncharacterized in most studies.

## The Aging Exposome: Hallmarks of Aging Captured in Past Studies?

Environmental pollution is considered as a major exposure affecting human health. The major sources of pollutions are particulate matter (PM), polycyclic aromatic hydrocarbons (PAHs), volatile organic compounds (VOCs), nitrogen, sulfur dioxides, carbon monoxide, ozone, and heavy metals. A quick browsing of extant literature suggested that studies on the effects of physical exposures such as sun (light), sound, noise, ultraviolet (UV) light, temperature on skin aging out number, the chemical exposure based aging studies.

In Figure 1B depicted is the progression of human aging from child birth (neonatal) through adolescence and the ultimate of life, death, that are riddled with exposure to chemicals surrounding us. As with incremental aging one experiences physiological decline, possibly as a result of the cumulative exposome that increases inside the human body. As this review was being submitted and peer-reviewed, an excellent new review was published that provided insights on molecular changes associated with exposures, causality, temporality in pathways leading to diseases, association of biological aging with socio-economic position, challenges with complex, multiple, and concurrent exposures and advancements in metabolomics and adductomics from a tool development perspective (Vineis et al., 2020). In the following sections, the existing literature on “aging exposome” have been thematically organized into several sub-sections purely based on the available content.

### The Skin Aging Exposome

The exposome resulting in skin aging is often attributed to pollution, solar radiation, smoking, temperature, nutrition, stress, and lack of sleep (Krutmann et al., 2017). The solar spectrum, comprised of ultraviolet light, can cause skin damage, including skin cancers, where atmospheric factors such as air pollution (i.e., smog, ozone, PM, etc.) are associated with premature skin aging (McDaniel et al., 2018). This review also noted that skin damage brought upon by environmental exposure is attributed to an array of chemical reactions initiated by elevated cellular levels of reactive oxygen species (ROS) in skin cells, which further causes oxidative insult to biomolecules such as proteins, lipids, and nucleic acids. Generally speaking, air pollution was shown to be a major exposure to have a large impact on human skin aging.

In Figure 1C, summarized are the sources of human chemical exposures that range from addiction (alcohol, drugs, cocaine, and smoking), to air pollution (particulate matter-PM, gases, and industrial vapors), water [pollutions, pesticides, per- and polyfluoroalkyl substances (PFAS) etc.], drugs (pharmaceuticals and medications), the resident internal and external gut microbiome, domestic waste, and indoor pollution (dust, cooking, and gases), occupational exposures (hospital disinfectants to asbestosis), pollution (fossil fuel burning to plastics), pets (carrying environmental pollution to microbiome), personal hygiene products (PHPs) (perfumes to soaps) to diet (and nutrition).

### Air Pollution Exposome in Aging

In 2018, the World Health Organization (WHO) recognized air pollution as the second-largest risk factor of non-communicable diseases, as more than 90% of global population breathe air quality that does not meet WHO definitions of clean air (Peeples, 2020). The US Environmental Protection Agency (EPA) defines particulate matter (PM), PM_10_ and PM_2.5_, as particles less than 10 and 2.5 μm in diameter, respectively (Peeples, 2020). Ambient atmospheric PM causes damage through generation of oxidative stress, that eventually leads to skin aging (Schroeder et al., 2006). More frequently, PM_2.5_ and PM_10_ have been more popularly studied in relation to human aging. In Table 1 listed are studies that associated various chemical exposures and their effect on aging in various human cohorts.

**TABLE 1:** List of studies that looked at exposure and its effect on aging in human studies.

Exposure	Cohort information	Findings	References
PM_2.5_	166 non-smoking elderly	5 μg/m^3^ increment in annual PM_2.5_ concentration was associated with a relative decrease of 16.8% (95% CI: –26.0%, –7.4%, *p* = 0.0005) in telomere length and a relative decrease of 25.7% (95% CI: –35.2%, –16.2%, *p* < 0.0001) in mtDNA content.	Pieters et al., 2016
PM_2.5_	Birth cohort study of 641 mother-new born pairs	Mothers with higher residential exposure; mothers gave birth to new-borns with significantly lower telomere length; a 5 μg/m^3^ increase in residential PM_2.5_ exposure during pregnancy, cord blood telomeres were 9% shorter and placental telomeres 13% shorter.	Martens et al., 2017
PM_2.5_ (indoor)	First group (*N* = 874) and a second examination group (*N* = 1003)	Indoor PM_2.5_ exposure levels were positively associated with skin aging symptoms, i.e., score of pigment spots on forehead (12.5% more spots per increase of IQR, *P* − 0.0371), and wrinkle on upper lip (7.7% more wrinkle on upper lip per increase of IQR, *P* − 0.0218).	Ding et al., 2017
PM_2.5_ component species (ammonium, elemental carbon, organic carbon, nitrate, and sulfate)	552 participants from the Normative Aging (yr. 2000–2011) (*n* = 940 visits)	Interquartile range increases in both 1-year sulfate (95%CI: 0.28, 0.74, *P* < 0.0001) and ammonium (95%CI: 0.02, 0.70, *P* = 0.04) levels were associated with at least a 0.36-year increase in Horvath DNAm-age; sulfate and ammonium were most associated with Horvath DNAm-age and suggest that DNAm-age measures differ in their sensitivity to ambient particle exposures and potentially disease.	Nwanaji-Enwerem et al., 2017
Air pollution (PM_10_, PM_2.5_, PM_2.5_ absorbance/black carbon (BC), and NO_*x)*_	KORA F4 cohort	BC and PM_10_ were broadly associated with biological aging in men; long-term exposure to air pollution is associated with biological aging measures, potentially in a sex-specific manner.	Ward-Caviness et al., 2016
Indoor pollution	Pingding (in northern China): *N* = 405; in Taizhou (in southern China) *N* = 857 women between 30 and 90 years of age	cooking with solid fuels was significantly associated with a 5–8% more severe wrinkle appearance on face and an 74% increased risk of having fine wrinkles on back of hands in both studies combined, independent of age and other influences on skin aging.	Li et al., 2015
BC	Male participants of the Normative Aging Study in the greater Boston, MA, United States	Association between BC and blood markers were not observed in main effects models or when stratified by obesity status; BC was positively associated with markers of inflammation in men with CHD (particularly vascular endothelial growth factor) and in men with diabetes (particularly interleukin-1β and tumor necrosis factor-α).	Fang et al., 2012
Smoking	966 individuals who participated in a case-control study to investigate environmental and genetic risk factors for skin cancer	Association among increasing age, sun exposure, and amount of telangiectasia was strong among men, but less apparent among women; smoking was also associated with elastosis among both sexes, and with telangiectasia predominantly among men.	Kennedy et al., 2003
Smoking	Spanish population, 1,474 participants (18–60 years old)	Most participants had Fitzpatrick skin phototype II (44.1%) and skin aging in accordance with their current age (69.0%); age, smoking habit, use of sunscreen and use of cosmetics were all significant independent predictors of skin aging.	Buendía-Eisman et al., 2020
Lead	459 men, Normative Aging Study, Boston	Blood lead concentration was positively and significantly associated with concurrent concentration of serum creatinine (*P* = 0.005); a10-fold increase in blood lead level predicted an increase of 7 μmol/L (0.08 mg/dL) in serum creatinine concentration; demonstrated impaired renal function in middle-aged and older men.	Kim et al., 1996
Lead	744 men, Normative Aging Study of the Department of Veterans Affairs	Rate of creatinine clearance was significantly and negatively associated with increasing levels of blood lead.	Payton et al., 1994
Lead	777 male participants (August 1991 and October 1996) in the Department of Veterans Affairs Normative Aging Study	A positive association between patellar bone lead and uric acid levels (*P* = 0.02); 52 (6.7%) participants had developed gouty arthritis; neither bone nor blood lead levels predicted gout in this cohort.	Shadick et al., 2000
Anaesthesia	1819 subjects with median (25th and 75th percentiles) follow-up of 5.1 (2.7–7.6) year and 4 (3–6) cognitive assessments	In older adults, exposure to general anaesthesia and surgery was associated with a subtle decline in cognitive *z*-scores.	Schulte et al., 2018
Solvents	41 floor layers and 40 carpenters	Among the oldest subjects (>60 years), only floor layers showed decline in visual memory; most highly exposed floor layers deteriorated significantly more than their referents in visual memory and perceptual speed.	Nilson, 2002

**Studies have mostly focused on PM_2.5_ (indoor and outdoor), smoking, lead (Pb), and other exposures.**

Air pollution exposure is also correlated with signs of skin aging such as pigment spots and wrinkles (Vierkötter et al., 2010). Increased soot and particles from traffic were associated with 20% higher pigment spots on forehead and cheeks (Vierkötter et al., 2010). Exposure to sun and smoking leads to enhanced risk for developing wrinkles than those in non-smokers and/or with reduced exposure to sun (Yin et al., 2001). Another study that explored the effect of PM_2.5_ (indoor air pollution) on two groups of individuals revealed that PM_2.5_ exposure levels were positively associated with skin aging, i.e., scores based on pigment spots on forehead and wrinkles on upper lip (Ding et al., 2017). Another study that investigated 522 participants of the Normative Aging cohort demonstrated that out of the PM_2.5_ component species, sulfate and ammonium were associated with at least a 0.36-year increase in Horvath DNAm-age (i.e., a measure of DNA methylation based age assessment) (Nwanaji-Enwerem et al., 2017). Other *in vitro* studies have shown that matrix metalloprotease I (MMP-1) expression was increased in fibroblasts upon exposure to either tobacco smoke extract or UVA. Moreover, PM, soot and nitrogen dioxide (NO_2_) have been associated with skin aging in several studies as reviewed elsewhere (Schikowski and Hüls, 2020). Further, epidemiological findings have suggested that the associations of PM can be altered by UV radiation, and provided evidence for an association of tropospheric ozone (O_3_) with wrinkle formation, independent of NO_2_, PM, and UV (Schikowski and Hüls, 2020). Currently, several research efforts are focussed at using untargeted chemical/exposomic profiling of the airborne organic exposome.

In the German KORA (Cooperative Health Research in the Region of Augsburg) F4 cohort, a follow-up study of the KORA S4 cohort (a “population-based” health survey conducted in the city of Augsburg and two surrounding counties between 1999 and 2001), it was shown that absorbance/black carbon (BC) and PM_10_ are associated with biological aging in men (Ward-Caviness et al., 2016). Further, in the Normative Aging Study conducted in the greater Boston, United States that consisted of male participants, revealed a positive association between BC and markers of inflammation in men with coronary heart disease (CHD) and diabetes (Fang et al., 2012). In the Washington Heights Inwood Community Aging Project (WHICAP), a prospective study of aging and dementia conducted with 4821 participants, higher concentrations of ambient air pollution [i.e., NO_2_, fine (PM_2.5_), and coarse (PM_10_)] were associated with rapid cognitive decline, and this association was higher in APOE-ε4 carriers (Kulick et al., 2020a). The same WHICAP cohort with 5,330 participants revealed that an increase in NO_2_ was associated with lower global cognitive score and its rapid decline (Kulick et al., 2020b). Moreover, aging and neurotoxicant induced neurodegeneration associated miRNAs include, miR-34 (targets genes such as P53, SIRT1, and PARKIN) miR-29 (targets VDAC1, P53, and BACE1) and miR-126 (targets Insulin/IGF-1/PI3K/AKT associated pathways) (Singh and Yadav, 2020), indicating miRNA-based regulation of neurotoxicant induced aging. Indoor pollution studied in two cohort of women from Northern and Southern China (30–90 years age, *N* = 1,262) revealed that cooking with solid fuels was associated with development of severe wrinkles on face and an increased risk of having fine wrinkles on back of the hands (Li et al., 2015). Cigarette smoking has been highly correlated with high rates of lung cancer from an analysis that leveraged associations between 2162 environmental exposures and lung cancer mortality rates in 2067 counties in the US (Juarez et al., 2017). A recent study that looked at the lung transcriptome in cigarette smoke-exposed mice predicted premature aging as indicated by gene expression patterns that showed strong upregulation of immunoglobulin genes in response to increased age and smoke exposure (Choukrallah et al., 2020). With increased global pollution (both indoor and outdoor), anthropogenic activities, and global climate change, skin aging might be accelerated as catalyzed by the deleterious chemical exposome.

### Effect of Exposome on Aging: Telomere Length

Telomere length attrition is an important hallmark of cancer. Numerous reviews have shown that exposure to air pollution and low residential green space exposure are associated with shortened telomere length (Martens and Nawrot, 2016). Further, particulate air pollution exposure can affect telomere mitochondrial axis of aging to display chronic health effects of air pollution, the telomere maintenance (and length) was proposed as a proxy for assessing the exposome (Martens and Nawrot, 2016). The environmental and occupational exposures associated with shorter telomere length are traffic-related air pollution (i.e., PM, BC, benzene and toluene), polycyclic aromatic hydrocarbons (PAHs), N-nitrosamines, pesticides, Pb, and deleterious waste exposure (Zhang et al., 2013). However, arsenic (As), persistent organic pollutants (POPs) and short-term exposure to PM were associated with longer telomere length (Zhang et al., 2013). Residential green space, lower traffic exposure, and long-term lower exposure to PM were associated with longer telomeres in children, young adults, and older individuals (Martens and Nawrot, 2018). Occupational exposures such as toxic metals, PAHs, and PM were associated with decreased telomere lengths for a given age, and a more recent exposure was associated with longer telomeres (Martens and Nawrot, 2018). For instance, 5-μg/m^3^ increment in annual PM_2.5_ concentration was associated with a relative decrease of 16.8% in telomere length and a relative decrease of 25.7% in mtDNA (mitochondrial DNA) content in an elderly population (*n* = 166, non-smokers) (Pieters et al., 2016). PAH-exposure led to reduced telomere length in peripheral blood cells in male non-drinkers when compared for PAH-exposed workers and controls (Bin et al., 2010). Bisphenol A (BPA) levels were strongly associated with senescence, inflammation, and were negatively correlated with the length of telomeres in patients with T2DM (*n* = 30) (Soundararajan et al., 2019). In a multicentre European birth cohort study HELIX (Human Early Life Exposome), reduced exposures to air pollution during pregnancy and childhood were associated with longer telomeres in children in Europe (Clemente et al., 2019). In the same HELIX cohort it was shown in another study that early life tobacco exposures via maternal smoking (measured as maternal urinary cotinine levels) during pregnancy may induce biological aging from early age as indicated in the shortened telomere lengths in the off springs (Osorio-Yáñez et al., 2020). In another study (166 non-smoking elderly participants) the increases in PM_2.5_ concentrations were associated with decrease in telomere length and a relative decrease in mitochondrial DNA (mtDNA) (Pieters et al., 2016). In a birth cohort study (*n* = 641, mother-new-born pairs) revealed that mothers with increased residential PM_2.5_ exposure gave birth to new-borns with significantly lower telomere length (reduced by 9 and 13% in cord blood and placental telomeres, respectively) (Martens et al., 2017). Another recent study that probed the combined effect of PAHs and phthalates exposure on telomere length and lung function in a pilot study conducted during the winter of 2014 and summer of 2015 in Wuhan city, China, revealed that 8 urinary monohydroxylated-PAHs (OH-PAHs) showed an overall effect on telomere length or lung function (Hou et al., 2020). When a study consisting of coke-oven workers (*n* = 1,005, males) looked at the association of PAH exposure and mosaic loss of chromosome Y (mLOY), one of the most common structure somatic event contributing to disease and mortality, found that a 10-fold increase in urinary 1-hydroxynaphthalene (1-OHNa), 1-hydroxyphenanthrene (1-OHPh), 2-OHPh, 1-hydroxypyrene (1-OHP) resulted in increased incidence of mLOY in a dose-dependent linear association (Liu et al., 2020). In another study that monitored healthy women residing in the Cape Town region of South Africa revealed that levels of personal NO_2_ and benzene exposure was inversely associated with leukocyte telomere length, where the magnitude of effects corresponded to > 6 year increase in chronological age (Everson et al., 2020). For a more recent update on air pollution and skin aging the readers are highly suggested to consult a recent review (Schikowski and Hüls, 2020). Evidence from these aforementioned studies and those provided in Table 1 indicate that reductions in air pollution (i.e., traffic and fossil fuel burning related), and environmental exposures (i.e., BPA, PAHs) may promote molecular longevity, as exemplified by telomere length, starting with neonates.

### Heavy Metals Accelerate Human Aging

Several heavy metals and their derivatives are implicated in accelerating human aging. Of the limited number of studies available, lead (Pb), aluminum (Al), and other heavy metals are reported to be associated with human aging. Pb, is known to affect the aging brain, and is associated with neurodegenerative diseases such as Alzheimer’s disease, and Parkinson’s disease, in addition to its ability to impact the epigenome (i.e., DNA methylation, histone modifications) and miRNA expression across diverse organisms (Eid and Zawia, 2016). Various studies suggest the role of Al in hastening brain aging resulting in increased incidence of specific age-associated neurological ailments (Bondy, 2014). Another study conducted in rats showed that neonatal exposure to Pb resulted in an enhanced expression of the β amyloid (Aβ) precursor protein (APP) gene, increased levels of APP and its proteolytic product, the Aβ peptide, and oxidative DNA damage in the aging brain (Basha, 2005; Bolin et al., 2006). A study on primate brains exposed to Pb as infants revealed that early life exposure to Pb reprogrammed the gene expression resulting in both up and down-regulation of genes through alternate epigenetic pathways contributing to an enhanced neurodegeneration in old age (Bihaqi et al., 2011). Another study conducted on 777 male participants (between August 1991 and October 1996) as part of the Department of Veterans Affairs Normative Aging Study revealed a positive association between patellar bone Pb levels and uric acid levels where 6.7% participants developed gouty arthritis (Shadick et al., 2000). Another study conducted on the Normative Aging Study of the Department of Veterans Affairs (*n* = 744 men) revealed that the creatinine clearance rate was negatively associated with increasing blood Pb levels (Payton et al., 1994). Another recent study that performed metabolomics on plasma samples from 399 men with measured Pb exposure in the VA Normative Aging Study cohort revealed that out of 858 metabolite quantified, 154 (17.9%) metabolites showed significant association with blood Pb levels (Kelly et al., 2020), where benzoate metabolism associated with long-term Pb exposure as assessed by toenails. In another recent study on 683 elderly men in the Normative Aging Study (conducted between 1999 and 2013), looking at DNAm PhenoAge acceleration (DNAmPhenoAccel), a new epigenetic biomarker of phenotypic age revealed that Pb and calcium (Ca) were significantly associated with DNAmPhenoAccel, whereas PM_2.5_, Pb, and silicon (Si) were predictors for DNAmPhenoAccel (Wang et al., 2020b). In a study on Canadian Study of Health and Aging (CSHA), a prospective cohort of 10, 263 subjects followed-up from 1991-1992 to 2001–2002 were probed for the association between Al in drinking water and Alzheimer’s disease but did not find a clear association between them (Van Dyke et al., 2020). In summary, based on the limited observations to discern the effect of heavy metals as an exposure on human aging needs more mechanistic studies on human metabolism, ethnicities, and through multiple model systems to ascertain the exact role heavy metals play in human health.

### Addiction (Alcohol, Smoking, and Drugs) and Other Exposures Affecting Human Aging

Addictions are another group of exposures that are chemically diverse, such as ethanol, cocaine, and nicotine. Smoking history decreases a human lifespan on average by 7 years (Bernhard et al., 2007), as smokes from cigarettes are known to contain > 4,000 toxic chemical compounds. Alcohol consumption has been associated with the higher DNA formamidopyrimidine DNA glycosylase (FPG)-sensitive sites, and only in the younger group of healthy women (i.e., > 40 years age) (Gonçalves Mota et al., 2017) (*P* < 0.05). Smoking as an exposure was associated with increasing telangiectasia and elastosis of skin in a study on 966 individuals investigated for environmental and genetic risk factors of skin cancer (Kennedy et al., 2003). Smoking habit was shown to be a significant independent predictor of skin aging in a Spanish population (*n* = 1,474 participants, 18–60 years old) (Buendía-Eisman et al., 2020). For occupational exposures, solvents used in carpentry can affect the aging process via changes in cognitive status when compared to unexposed individuals. Older subjects (i.e., carpenters) that were > 60 years old showed significantly high deterioration in visual memory and perceptual speed (Nilson, 2002). Among other life exposures, in older subjects (*n* = 1,819) exposed to hospital procedures such as general anaesthesia and surgery revealed a subtle decline in cognition (Schulte et al., 2018). Even biological exposures such as viral infections, i.e., human immunodeficiency virus (HIV) and cytomegalovirus (CMV) affect an aging immune system (Leng and Margolick, 2020). In summary, exposures that negatively affect aging can be chemically diverse, ranging from those originating with recreation or addition to those associated with a disease or its drug treatment.

### Chemoprotection From Aging

There are also exposure chemicals that are known to be protective against progressive human aging- and used either as desirable or undesirable substances. For instance, rats fed a diet with 0.06 or 0.6 ppm of selenium (Se) as sodium selenite enriched diet, revealed that Se increased running in unexposed animals as they aged, and protected them against methylmercury (MeHg) (provided in drinking water for 16 months at 0.5, 5.0, or 15 ppm doses of Hg) induced neurotoxicity (Heath et al., 2010). Also, crocin, from saffron plant (a dietary component in the form of a spice), displayed ROS amelioration, and protected cellular squalene from UVA-induced peroxidation (Fagot et al., 2018).

**FIGURE 2:**
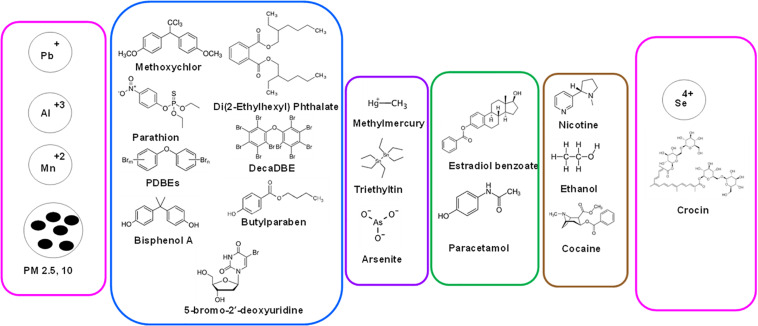
Range of human chemical exposures that are associated with aging. From heavy metals/ions to particulate matter (PM) of various sizes (PM_2.5_, PM_10_), carbon black (BC), pesticides, PDBEs, phthalates, BPA, drugs (methamphetamine, paracetamol) to metalloids (methylmercury, arsenate) to chemicals from addiction (ethanol, nicotine, cocaine). Shown also are selenium and crocin (from Saffron plant) that exert protective effect on aging.

In Figure 2, summarized are human chemical exposures that have been associated with aging- heavy metals/ions, particulate matter (PM) of various sizes (PM_2.5_, PM_10_), BC, pesticides, PDBEs, phthalates, BPA, drugs (methamphetamine, paracetamol), metalloids (methylmercury, arsenate) and chemicals from addiction (ethanol, nicotine, and cocaine).

## Evidence From Exposome-Aging Association Studies Conducted in Animal Models

Experimental animal model systems have always provided meaningful mechanistic insights into the processes of biological aging as well as for understanding of the physiological responses of an exposure to aging in humans. Simple exposure to low-dose estradiol during development and to the endocrine disruptor chemical (EDC), BPA result in increased susceptibility to prostatic intraepithelial neoplasia (PIN) lesions with aging upon cumulative adult exposure to estradiol (Prins et al., 2007). Very recently, using a rat model of exposure to an EDC, it was demonstrated that early-life chemical exposure caused metabolic dysregulation in adulthood as evident from the reprogrammed histone signatures in the developing liver (Treviño et al., 2020). In this study it was demonstrated that EDC exposure hijacked age-related epigenomic plasticity to accelerate epigenomic aging. Moreover, it was Horvath who first proposed the “epigenetic clock” (i.e., DNA methylation (DNAm)-based estimates of age), that provided an precise age prediction based on the total methylation levels of multiple CpG loci (Horvath et al., 2015). Among other models, a recent study conducted in zebrafish (*Danio rerio*) model for cannabidiol (CBD) exposure revealed significantly enhanced survival and reduced size of female fish and reduced sperm concentration in male fish (Pandelides et al., 2020). Table 2 provides a list of studies that investigated exposures and their effects on aging in animal models and through *in vitro* studies. The following sections cover the findings from multiple model systems in age- chemical exposure association studies.

**TABLE 2:** List of studies that looked at exposure and its effect on aging in animal models.

Exposure	Models	Treatment	Findings	Link
Fatty acid hydroperoxides (OFA) and/or vit E	Porcine cells	Exposure to 5 μM OFA for 40 passages	Age-related decrease in **endothelial barrier function (EBF),** while supplementation of OFA-treated cultures with 25 μM vit E protected against the OFA-mediated decrease in EBF, chronic oxidative stress decreased EBF, predisposing the artery to infiltration by blood components and subsequent atherogenesis.	Boissonneault et al., 1990
Bisphenol A (BPA)	*C. elegans*	Does of 0, 100, 500, and 1,000 μM of BPA, and 0, 10, 25, and 50 μM of BPA	Increased the generation of **hydrogen peroxide-related ROS** and **superoxide anions**, accelerated aging by the induction of oxidative stress.	Tan et al., 2015
Arsenite	*C. elegans*	Arsenite exposure (100 μM)	Age biomarkers affected: a decrease **of defecation frequency**, accumulations of **intestinal lipofuscin and lipid peroxidation** in an age-dependent manner; increased **intracellular ROS** level; increased mRNA levels of transcriptional makers of aging (*hsp-16.1*, *hsp-16.49*, and *hsp-70*); chronic arsenite exposure resulted in accelerated aging process.	Yu et al., 2016
Arsenite	*C. elegans*	Exposure to 0.1–1,000 μM arsenite	Transient **increase in ROS levels** in, co-exposure to ROS scavengers prevented the lifespan-extending capabilities of arsenite (i.e., mitohormesis), low-dose arsenite extended lifespan, providing evidence for nonlinear dose-response characteristics of toxin-mediated stress resistance and longevity.	Schmeisser et al., 2013
Cocaine, nicotine	*Rattus norvegicus*	High-dose cocaine (40 mg/kg/d), or, high-dose nicotine (5.0 mg/kg/d)	Prenatal exposure showed long-term effects on **emotional behavior**. Combinations led to **depressive symptoms**, in a dose-dependent manner.	Sobrian et al., 2003
Alcohol	*Rattus norvegicus*	Liquid alcohol diets containing 0, 17.5, or 35% ethanol-derived calories, from gestation d 7 to parturition	Showed a **peripheral auditory disorder** in the form of congenital sensorineural hearing loss; punctate lesions and malformed stereocilia on the auditory sensory receptor cells of the inner ear; central auditory processing disorder characterized by prolonged transmission of neural potentials along the brainstem portion of the auditory pathway; age-related deterioration of auditory acuity.	Church et al., 1996
Endocrine disruptors	*Rattus norvegicus*	Twelve consecutive d of vehicle (dimethylsulfoxide), estradiol benzoate (EB) (1 mg/kg), and MXC (methoxychlor) (low dose, 20 μg/kg or high dose, 100 mg/kg), beginning on embryonic d 19 through post-natal d 7	Early life exposure has lifelong effects on **neuroendocrine gene expression and DNA methylation**, caused advancement of **reproductive senescence**	Gore et al., 2011
Endocrine disrupting chemicals (EDCs)	*Rattus norvegicus*	Mixture of phthalates, pesticides, UV-filters, bisphenol A, butylparaben, paracetamol	In pre-pubertal rats, a significant reduction in primordial follicle numbers, reduced plasma levels of prolactin; incidence of irregular estrous cycles was higher, reduced ovary weights, raises concern regarding potential effects of mixtures of EDCs on female reproductive function.	Johansson et al., 2016
Estradiol benzoate (EB)	*Rattus norvegicus*	Silastic implants containing estradiol benzoate (EB) in solution placed into 5 d old female Wistar rats and removed 1 or 5 d later	Premature occurrence of vaginal opening was observed in all three estrogenized groups independently of EB; ovaries of these last two groups showed a reduced number of corpora lutea and an increased number of large follicles, ovarian weight and progesterone content gradually.	Rodriguez et al., 1993
Methylmercury	*Rattus norvegicus*	Pregnant females exposed to drinking water containing 0, 0.5, or 6.4 ppm Hg as methylmercury, i.e., 40 and 500 μg/kg/d of mercury intake	Prenatal exposure **retarded the acquisition of choice** in older rats, yet, two rate measures, lever-press rates and changeover rates, **were not systematically affected** by methylmercury.	Newland, 2004
Methylmercury	*Rattus norvegicus*	Doses of 0, 0.5, or 6.4 ppm Hg in the drinking water of female rats at least 4 weeks before mating and continuing until post-natal 16 d	Gradual decline in the reinforcement rate began to appear in low- and high-dose rats at about 500 and 800 d of age, respectively, the lever-press duration increased, the inter-response time (IRT) was unaffected, and the time between response bursts increased.	Newland and Rasmussen, 2000
Triethyltin (TET)	*Rattus norvegicus*	Dosed with TET (5 mg/kg)	At 24 months, TET-treated rats showed significant deficits in **acquisition and retention of the water maze task;** resulted in morphological damage that was apparent behaviourally.	Barone et al., 1995
Aluminum	*Rattus norvegicus*	Pregnant females orally exposed to 0, 50, and 100 mg Al/kg/d	Decreased learning performance of the task in both adult and old rats, age-related effect on water maze performance, accumulation of Al in brain at 2 years of age.	Roig et al., 2006
5-bromo-2’-deoxyuridine	*Rattus norvegicus*	Subcutaneous injections (3.2 mg) of a synthetic analog of thymidine, 5-bromo-2’-deoxyuridine (BrdUrd), on 1 and 3 d, or 1, 3, 7, and 21 d of post-natal life	Mean life span decreased by 31 and 38% in male and by 14 and 27% in female rats that received 2 and 4 injections of BrdUrd; opening of the vagina was delayed, inhibition of compensatory ovarian hypertrophy induced by hemiovariectomy at the age of 3 months was found in females; neonatal administration of BrdUrd to rats doubles the incidence of chromosome aberrations in peripheral blood lymphocytes; dose-related increase in tumor incidence.	Anisimov and Osipova, 1992
Di(2-Ethylhexyl) Phthalate	*Mus musculus*	Orally dosed DEHP (20μg/kg/d−500 mg/kg/d) daily for 10 d	DEHP significantly decreased inhibin B levels; significantly increased the BAX/BCL2 ratio in primordial follicles leading to a significant decrease in primordial and total follicle numbers	Hannon et al., 2016
DecaBDE Polybrominated diphenyl ethers (PBDEs)	*Mus musculus*	Daily oral dose of 0, 6, or 20 mg/kg decaBDE from post-natal 2–15 d	Performance of the aging cohort was significantly affected, on the light–dark discrimination, older decaDBE treated mice learned the task more slowly, made more perseverative errors after an initial error, and had lower latencies to respond compared with controls.	Rice et al., 2009
Alcohol	*Mus musculus*	Single teratogenic dose (5.8 g/kg) of ethyl alcohol during organogenesis on the 9^*th*^ d of gestation	In adulthood, the offspring suffered a **deficit in long-term retention**, but not acquisition, of a place learning task; the **retention deficit** was severe in aging mice.	Dumas and Rabe, 1994
Lead	*Mus musculus*	Pb/E exposure, 0.2% Pb-acetate added to the deionized drinking water of the pregnant female	**Up-regulation of genes** related to the immune response, metal-binding, metabolism and transcription/transduction coupling; disturbances in developmental stages of the brain compromise the ability to defend against age-related stressors.	Dosunmu et al., 2012

### *In vitro* Cellular Model Systems

Mammalian cell culture models offer a time-proven quick *in vitro*, high throughput, inexpensive assay and screening capabilities for bioactivity screening of exposures, and have helped obtain enormous insights into *in vivo* metabolism of chemical exposures, though with all sorts of limitations of the system. Diverse studies have been performed to study the mechanisms of action and toxicity of chemical exposures, though studies w.r.t aging are scarce. In one such exposure study in *in vitro* cell models, when 5 μM of fatty acid hydroperoxides (OFA) were exposed to cells for 40 passages, age-related decrease in endothelial barrier function (EBF) was observed, predisposing the artery to infiltration by blood components and eventual atherogenesis (Boissonneault et al., 1990).

### *Caenorhabditis elegans* Models

The model organism, *Caenorhabditis elegans* has been widely used to screen the effects of diverse chemical exposures over the years for the amenability and simplicity of this model system among other reasons. The *C. elegans* model has lend itself as a top model in developmental toxicity studies (Boyd et al., 2012) and has helped provide mechanistic insights on toxicity of POPs and EDCs (Chen et al., 2019). With *C. elegans* already a well-established model of aging (Johnson, 2008; Tissenbaum, 2015; Denzel et al., 2019), it offer the benefits of using this model system for studying individual/multiple chemical exposures w.r.t to aging research. Assays performed using chemotherapeutic agents and environmental compounds from ToxCast Phase I library helped compare results against the mammalian *in vitro* end point data from ToxRef database (DB) with regards to germline function (Allard et al., 2013). Further, several medium-throughput (feeding, growth, reproduction, and locomotion) and two high-throughput (growth and reproduction) assays have been developed in *C. elegans* to decipher the effects of potential neurotoxicants (Boyd et al., 2010b). Moreover, several chemical exposures such as chlorpyrifos (Roh and Choi, 2008), dibutyl phthalate (DBP), a likely endocrine disruptor and frequently used plasticizer, and the pesticides 2-(thiocyanomethylthio) benzothiazole (TCMTB) and permethrin (Shin et al., 2019), 1-alkyl-3-methylimidazolium chloride ionic liquids (Swatloski et al., 2004), heavy metal cadmium (chloride) (Boyd et al., 2010a) and arsenite (Wang et al., 2007), chemical repellents such as quinine, denatonium, detergents, other heavy metals (Hilliard et al., 2005), natural estrogens, 17β-estradiol (E2), and environmental pollutants such as bisphenol A (BPA) and tributyltin chloride (TBTCL) (Hoshi et al., 2003), triclosan (TCS) and triclocarban (TCC) (Lenz et al., 2017), nanopolystyrene (Qiu et al., 2020) among others. BPA exposure was shown to increase the generation of hydrogen peroxide-related ROS and superoxide anions, further accelerating the aging process by inducing oxidative stress (Tan et al., 2015). Two studies that probed arsenite exposure on *C. elegans* revealed a reduced amount of defecation, increased intestinal lipofuscin. and lipid peroxidation with age alongside increased intracellular ROS levels (Yu et al., 2011). Further, co-exposure to ROS scavengers prevented the lifespan-extending capabilities of As (i.e., mitohormesis) (Schmeisser et al., 2013). These studies indicate that *C. elegans* will continue to lead the path in studying both exposomics and aging in the same model.

### Rat Models

As a simpler mammalian model system, rats have been used extensively in research encompassing chemical exposures over the past few decades. Rats showed long-term effects on emotional behavior that led to depressive symptoms when exposed to prenatal high doses of both cocaine and nicotine (Sobrian et al., 2003). Alcohol (ethanol) exposure in rats (during gestation to parturition) showed a peripheral auditory disorder, i.e., congenital sensorineural hearing loss and age-related deterioration of auditory acuity (Church et al., 1996). Rats exposed to endocrine disruptor chemicals (EDCs) such as estradiol benzoate (EB) and MXC (methoxychlor) in early life affected neuroendocrine gene expression, and caused advancement of reproductive senescence (Gore et al., 2011). Exposure to a mixture of EDCs such as, phthalates, pesticides, UV-filters, BPA, butylparaben, paracetamol in pre-pubertal female rats led to a significant reduction in primordial follicle numbers, reduced plasma levels of prolactin, higher incidence of irregular oestrous cycles, and reduced ovary weights (Johansson et al., 2016). Exposure of estradiol benzoate in young female rats led to premature occurrence of vaginal opening, decreased number of corpora lutea, and an increased number of large follicles (Rodriguez et al., 1993).

Several other studies have focussed on the exposure of rats to heavy metals and organometallics. Pregnant female rats (prenatal) exposed to methylmercury (CH_3_.Hg) slowed the acquisition of choice in older rats (Newland, 2004), whereas a temporal reduction in the reinforcement rate began to appear in low- and high-dose rats (Newland and Rasmussen, 2000). Rats exposed to triethyltin (TET) revealed reduced levels of acquisition and retention of the water maze task as well as morphological damages (Barone et al., 1995). Al exposure resulted in decreased learning performance of the task in both adult and old rats, and an age-related effect on water maze performance (Roig et al., 2006). Rats exposed to synthetic analog of thymidine, 5-bromo-2′-deoxyuridine (BrdUrd) resulted in the following: mean life span decreased significantly, where in female rats the opening of the vagina was delayed, doubled the events of chromosomal aberrations in blood lymphocytes and showed dose-related increase in tumor incidence (Anisimov and Osipova, 1992). Thus, the rat models continue to provide mechanistic insights in response to chemical exposures in laboratory set ups.

### Mice Models

Mice models have been used to measure the effects of chemical exposures with respect to aging. Mice orally exposed to di(2-Ethylhexyl) phthalate showed significantly decreased levels of inhibin B, enhanced the BAX/BCL2 ratio in primordial follicles resulting in reduced total number of follicles (Hannon et al., 2016). Mice exposed to decaBDE, a specific polybrominated diphenyl ethers (PBDEs) class chemical, demonstrated a reduction in performance such as light–dark discrimination and learning tasks in older decaBDE treated mice (Rice et al., 2009). Ethyl alcohol was shown to induce severe retention deficit and long-term retention in aging mice (Dumas and Rabe, 1994). Exposure to Pb in female pregnant mice resulted in up-regulation of transcripts related to the immune response, metal-binding, metabolism and transcription/transduction coupling (Dosunmu et al., 2012). Piperine, an alkaloid nutrient component of *Piper nigrum*, when exposed to a D-galactose-induced aging mouse model ameliorated the D-galactose induced cholinergic malfunction, extensive oxidative stress, neuroinflammation, and hyperphosphorylation of tau protein in hippocampus of these animals (Wang et al., 2020a). Based on the limited studies, one can clearly conclude that the mice models can offer a system to screen single or multiple chemical exposures in various strains that are yet to be explored.

## Tools and Techniques to Capture the Exposotypes

### Analytical Platforms to Capture Exposome

Generally speaking, past studies have typically focused on measuring the chemical space (i.e., metabolome and now, exposome) in human samples and habitats using spectrometry tools such as mass-spectrometry (MS) hyphenated with diverse chromatography approaches as mentioned elsewhere (Misra, 2019). Primarily these include, gas chromatography (GC)-MS, liquid chromatography (LC)-MS, capillary electrophoresis (CE)-MS etc. or without chromatography, i.e., direct injection (DI)-MS, flow injection analysis (FIA)-MS, imaging-MS and spectroscopy approaches such as nuclear magnetic resonance spectroscopy (NMR), Raman spectroscopy, infra-red (IR) spectroscopy etc. Other MS-based technologies are equipped to provide increased overall measurement dynamic range of environmental chemicals from polar to highly non-polar chemicals from a wide variety of matrices (Misra, 2019).

### Software Tools, Databases, and Resources to Annotate Exposome

Over the past decade, there have been explosions in cheminformatics approaches enabling deep data mining efforts in the exploration of human exposome. Both, spectral databases and chemical structure/formula databases have enabled identification of novel compounds in diverse samples/matrices of environmental, biological, and human origin. Improvement of chemical structure databases have enabled development of bioinformatics tools for chemical dereplication and/or annotation of true “unknown unknown” structures. Currently, Mass Bank of North America (MoNA) has collected ∼6.5 million spectra (of which 1.3 million are MS/MS spectra) and the METLIN DB now has data for over 800,000 MS/MS spectral data collected at multiple energies that are accessible. From a data stand point, there are still gaps in the community standards (for exposomics data sharing and archiving), and lacks an active or implemented Minimum Information About an Exposomics Experiment (MIAEEE) (Misra, 2016). In Table 3, listed are current software tools, resources, and DBs to analyze the captured exposome from various analytical platforms.

**TABLE 3:** List of software tools, resources and chemical and spectral databases for exposomics research.

Tool	Link/Availability	Type	Utility/Description	References
**Software tools**				
*BioTransformer*	http://biotransformer.ca/	Webserver	*-In silico* prediction of metabolism (biotransformations), compound identification - A machine learning and knowledge-based approach to predict small molecule metabolism in human tissues (e.g., liver), human gut and environment	Djoumbou-Feunang et al., 2019
Competitive Fragmentation Modeling (CFM)-ID	http://cfmid.wishartlab.com/	Webserver	-Performs annotation of peaks in a spectrum for a known chemical structure; predicts of spectra for a given chemical structure and putative metabolite identification (a predicted ranking of possible candidate) - Out-performs existing methods, i.e., MetFrag and FingerID structures for a given spectrum	Allen et al., 2014
*ChemDistiller*	https://bitbucket.org/iAnalytica/chemdistillerpython	Python	-Automated large-scale annotation of metabolites using tandem MS data -Interrogated against a compiled DB containing tens of millions of compounds with pre-calculated fingerprints and fragmentation patterns	Laponogov et al., 2018
*CSI (Compound Structure Identification): FingerID*	https://bio.informatik.uni-jena.de/software/sirius/	Standalone, Java	-Combines fragmentation tree computation and machine learning. -Finds 150% more correct identifications than the second-best search method	Dührkop et al., 2015
*InterpretMSSpectrum*	https://cran.r-project.org/web/packages/InterpretMSSpectrum/index.html	R	- Annotating and evaluating in-source mass spectra as obtained from full-scan experiments - Locates the molecular ion, fragment, and adduct peaks, calculates their most likely sum formula combination, and provides an annotated mass spectrum - Amenable for GC/APCI-MS data	Jaeger et al., 2016
*MetFrag*	http://c-ruttkies.github.io/MetFrag/	Web	-Combines compound DB searching and fragmentation prediction for metabolite identification from tandem MS data - Retrieval of reference, data source and patent information via ChemSpider and PubChem web services possible	Ruttkies et al., 2016
*MS2LDA*	http://ms2lda.org	Web	- Uses tandem MS data in many standard formats and allows the user to infer the sets of fragment and neutral loss features that co-occur together (Mass2Motifs). -In an alternative workflow, the user can also decompose a data set onto predefined Mass2Motifs	Wandy et al., 2018
*MS-FINDER*	http://prime.psc.riken.jp/Metabolomics_Software/MS-FINDER/index.html	Standalone	-Molecular formulas of precursor ions are determined from accurate mass, isotope ratio, and product ion information. -All isomer structures of the predicted formula are retrieved from metabolome DBs, and MS/MS fragmentations are predicted *in silico*. -Predicted structures are ranked by a combined weighting score considering bond dissociation energies, mass accuracies, fragment linkages, and nine HR rules	Tsugawa et al., 2016
*mzCloud*	https://www.mzcloud.org/	Web	- An extensively curated DB of HR tandem mass spectra that are arranged into spectral trees. -MS/MS and multi-stage MS^*n*^ spectra were acquired at various collision energies, precursor m/z, and isolation widths using Collision-induced dissociation (CID) and Higher-energy collisional dissociation (HCD). -Each raw mass spectrum was filtered and recalibrated giving rise to additional filtered and recalibrated spectral trees that are fully searchable.	NA
*SIRIUS 4*	https://bio.informatik.uni-jena.de/software/sirius/	Standalone	-Computational approach for molecular structure identification. -SIRIUS 4 integrates CSI:FingerID for searching in molecular structure DBs. -Can achieve identification rates of > 70% on challenging metabolomics datasets.	Dührkop et al., 2019
**Databases**				
*Blood Exposome DB*	http://exposome.fiehnlab.ucdavis.edu/	Web	-Consists of 41,474 achiral structures that were linked to 65,957 PubChem CIDs and to over 878,966 PubMed articles - Can help prioritize chemicals for systematic reviews, developing target assays in exposome research, identifying compounds in untargeted mass spectrometry, and biological interpretation in metabolomics data	Barupal and Fiehn, 2019
*ChemSpider*	http://www.chemspider.com/	Web	- An online chemical DB offering access to physical and chemical properties, molecular structure, spectral data, synthetic methods, safety information, and nomenclature for almost 25 million unique chemical compounds -Linked to almost 400 separate data sources on the Web.	Pence and Williams, 2010
*Exposome Explorer 2.0*	http://exposome-explorer.iarc.fr/	Web	-185 candidate dietary biomarkers with 403 associations with food intake (from metabolomic studies) -1,356 associations between dietary biomarkers and cancer risk in epidemiological studies, collected from 313 publications	Neveu et al., 2019
*FooDB*	http://foodb.ca/	Web	- Dietary macronutrients and micronutrients information, flavor, color, taste, texture and aroma. -Each entry with > 100 separate data fields covering detailed compositional, biochemical and physiological information -search amenable for browsing FooDB by food source, name, descriptors, function or concentrations.	NA
*MassBank*	https://massbank.eu/MassBank/	Web	-605 electron-ionization mass spectrometry (EI-MS), 137 fast atom bombardment MS and 9,276 electrospray ionization (ESI)-MS*^*n*^* data of 2,337 authentic compounds, 11 545 EI-MS and 834 other-MS data of 10 286 volatile natural and synthetic compounds, and 3,045 ESI-MS^2^ data of 679 synthetic drugs contributed by 16 research groups (as of January 2010).	Horai et al., 2010
*MoNA (MassBank of North America)*	http://massbank.us	Web	-Metadata-centric, auto-curating repository designed for storage and querying of mass spectral records. -DB of metabolite mass spectra, metadata and associated compounds. -contains over 200,000 mass spectral records from experimental and in-silico libraries as well as from user contributions	NA
*METLIN Exposome DB*	https://metlin.scripps.edu/	Web	-To aid identification of environmental toxicants, food contaminants and supplements, drugs, and antibiotics and their biotransformation products - > 700,000 chemical structures to now include more than 950,000 unique small molecules -XCMS/METLIN platform now allows for the readout of the biological effect of a toxicant through metabolomic-derived pathway analysis	Warth et al., 2017
*NIST Chemistry WebBook*	http://webbook.nist.gov	Web	-Consists of thermochemical, ion energetics, solubility, and spectroscopic data. - Thermochemical data available include enthalpies of formation, enthalpies of phase transitions, and heat capacities.	Linstrom and Mallard, 2001
*NMRShiftDB*	http://nmrshiftdb.nmr.uni-koeln.de	Web	-First NMR DB that allows open access to the DB and open and peer reviewed submission of datasets -Free repository of assigned ^1^H and ^13^C NMR spectra. -Collaborating laboratories can fully replicate the DB and to create a highly available network of NMRShiftDB mirrors. -DB contains about 10,000 structures and assigned spectra	Steinbeck and Kuhn, 2004
*Phenol-Explorer 3.0*	http://www.phenol-explorer.eu	Web	- Data on the effects of food processing on polyphenol contents in foods. - > 100 foods, covering 161 polyphenols or groups of polyphenols before and after processing (collected from 129 peer-reviewed publications)	Rothwell et al., 2013
*repoDB*	http://apps.chiragjpgroup.org/repoDB/	Web	-A gold standard DB, repoDB, that consists of both true positives (approved drugs), and true negatives (failed drugs)	Brown and Patel, 2017
*ReSpect* (RIKEN tandem mass spectral DB	http://spectra.psc.riken.jp/	Web	-Plant specific (phytochemical) MS/MS spectra DB/resource for fragment pattern analysis of tandem MS data - 3,595 metabolites in ReSpect, 76% were derived from 163 literature reports, rest were obtained from authentic standards. -A fragment search was established based on only the *m/z* values of query data and records.	Sawada et al., 2012
*SDBS* DB	https://sdbs.db.aist.go.jp/sdbs/cgi-bin/cre_index.cgi	Web	-An integrated spectral DB system for organic compounds, which includes 6 different types of spectra under a directory of the compounds. -The six spectra are as follows: an EI-MS spectrum, a Fourier transform infrared spectrum (FT-IR), a1HNMR, 13C NMR spectrum, a laser Raman spectrum, and an electron spin resonance (ESR) spectrum.	NA
*T3DB (Toxin and Toxin Target/Toxin Exposome) DB*	http://www.t3db.ca/	Web	-First version of T3DB (in 2010) contained data on nearly 2900 common toxic substances along with detailed information on their chemical properties, descriptions, targets, toxic effects, toxicity thresholds, sequences (for both targets and toxins), mechanisms and references -Latest release of T3DB includes more compounds (>3,600), targets (>2,000) and gene expression datasets (>15,000 genes). -Contains informative chemical ontologies and a large number of referential NMR, MS/MS and GC-MS spectra.	Wishart et al., 2015

Software tools such as BioTransformer can help to predict *in silico* metabolism of a metabolite/environmental chemical by following human metabolic pathway reactions that are gut, tissue or environment specific (Djoumbou-Feunang et al., 2019). Competitive Fragmentation Modeling (CFM)-ID can aid in annotation of spectral peaks, in prediction of spectra of a given chemical structure, and in putative identification of metabolites from unknown spectra (Allen et al., 2014). ChemDistiller can help annotate metabolites using tandem MS data against a compiled DB of millions of compounds (Laponogov et al., 2018). CSI (Compound Structure Identification): FingerID combined fragmentation tree computation and machine learning to help resolve unknown compound identification challenges (Dührkop et al., 2015). InterpretMSSpectrum is a tool suitable for annotating and evaluating in source full-scan mass spectra from gas chromatography-atmospheric pressure chemical ionization-mass spectrometry (GC/APCI-MS) data (Jaeger et al., 2016). MetFrag can perform compound DB searching and fragmentation prediction for metabolite identification of tandem MS spectral data (Ruttkies et al., 2016). MS2LDA uses tandem MS data for inferencing sets of fragment and neutral loss features that co-occur in spectral data for aiding in unknown interpretation (Wandy et al., 2018). Using tools such as MS-FINDER one can determine molecular formulas of precursor ions from accurate mass, isotope ratio etc. where the predicted formulae are retrieved from metabolome DBs and follow the nine hydrogen rearrangement (HR) rules (Tsugawa et al., 2016). mzCloud is a curated DB of HR tandem MS data arranged as spectral trees, where tandem MS and multistage MS^*n*^ data were acquired at various collision energies. SIRIUS 4 is another computational approach for molecular structure identification (Dührkop et al., 2019).

**FIGURE 3:**
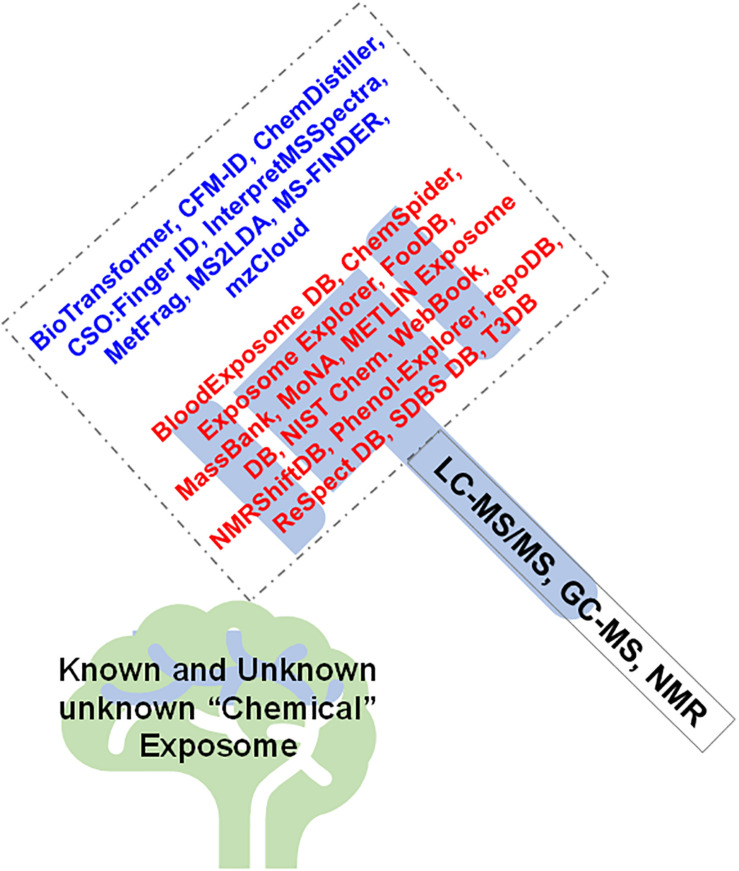
Mass spectrometry and spectroscopy provide the necessary analytical tools that aid in capturing the aging “exposome,” which when processed by the software tools, resources, and databases enable to “crack” (identify) the known and unknown chemical exposome.

There are several chemical and spectral DBs that aid in annotation of known exposomic chemicals, metabolites, as well as can help decipher the molecular formulae and structure of unknown chemicals. Leading the pack is the Blood Exposome DB that consists of 65,957 PubChem chemical IDs extracted from 878,966 PubMed article constituting all chemicals ever associated with blood as a biological matrix (including plasma, serum, and blood cell-types) (Barupal and Fiehn, 2019). ChemSpider is a resource linked to > 400 data sources on the web is an enormous chemical DB offering information on physio-chemical properties, molecular structure, spectral data, safety data and nomenclature for 25 million unique chemicals (Pence and Williams, 2010). Exposome Explorer 2.0 is a DB of exposomal chemicals, i.e., 185 dietary biomarkers with 403 associations obtained from food intake, and 1,356 associations between dietary biomarkers and cancer risk from epidemiological studies (Neveu et al., 2019). FooDB is a DB with information of dietary micro and macronutrients, with > 100 separate data fields for each chemical entry covering a chemical’s compositional, biochemical and physiological information. MassBank is a spectral DB with 100s of EI-MS, ESI-MS/MS, FAB-MS spectra for authentic metabolites, volatile and synthetic compounds, drugs etc. (Horai et al., 2010). MoNA is a mass spectral DB with 200,000 mass spectral records from experimental and *in-silico* libraries as well as from user contributions. METLIN Exposome DB can aid in identification of environmental toxicants, food contaminants, supplements, drugs, and their biotransformation products from > 950,000 unique chemicals. NIST Chemistry Webbook is a DB with thermochemical, ion energetics, solubility and spectrometric data (Linstrom and Mallard, 2001). NMRShiftDB is the first ever free repository of assigned ^1^H and ^13^C NMR spectra that contains > 10,000 structures and assigned spectra (Steinbeck and Kuhn, 2004). Phenol-explorer 3.0 is a DB with information on effects of food processing on polyphenol contents for > 100 foods, covering 161 polyphenols (Rothwell et al., 2013). RepoDB consists of information on approved and failed drugs as highly curated DB (Brown and Patel, 2017). ReSpect (RIKEN tandem mass spectral DB is a phytochemical tandem MS spectral DB that allows fragment pattern analysis from raw spectra based on the information content of the DB that has spectra from 3595 metabolites obtained from authentic standards (Sawada et al., 2012). SDBS DB is a spectral DB for organic compounds that has 6 different spectra types, i.e., EI-MS, FTIR, 1HNMR, ^13^CNMR, laser Raman and EST spectra. T3DB (Toxin and Toxin Target/Toxin Exposome DB) consists of compounds (>3,600), targets (>2,000) and gene expression datasets (>15,000 genes) and chemical ontologies and a large number of referential NMR, MS/MS and GC-MS spectra. For common toxic substances (Wishart et al., 2015). Other resources include such as the NORMAN Suspect List Exchange (NORMAN-SLE), that was established in 2015 for NORMAN members (and others) to find suspect lists relevant for their environmental monitoring^1^. Figure 3 displays the combined approach of using high throughput analytical platforms (i.e., LC-MS/MS, GC-MS, NMR, other spectroscopy) to enable capture the aging “chemical exposome,” that further uses the power of computation in exploiting the software tools, DBs and resources available to aid in identification of the known and unknown chemical exposures.

## Opportunities and Challenges in Studying the Aging Exposome: Unanswered Questions and Future Scope

A glance at the above literature from the past limited studies on the aging-exposome association, with scattered evidence from both animal models and human cohorts raise a handful of questions that warrants answers. Some of these questions are provided below:

1. How does one translate the exposome studies in cell lines, *C. elegans*, and mice models to human applications, given limitations of the models and at the same time challenges associated with human studies (i.e., an exposomal intervention study other than diet)?
2. What contributes to aging more- the genome or the exposome, both, or is it more dependent on the exposure in question?
3. If there is a need for exposome DB, then beyond the blood exposome DB, one needs to explore organ system (i.e., liver, kidney, gut exposome DBs) and model based exposome DBs to further our understanding of the human systemic exposome in the context of aging?
4. Can the international efforts in studying human aging from various dimensions (biochemical, ethnicities, pathophysiological, brain to gut microbiome studies) come together to collectively explore the “universal exposome signature of human aging”?
5. Do we need different and custom statistical models to associate aging with exposome (i.e., as exposures follow different regulations from genes, proteins, and metabolites that are endogenous) given that the exposome is cumulative, simultaneous, and interactive?
6. How would one decompose the biomolecular signatures of aging from those that are exposome mediated processes in human and other experimental models?
7. What could be the impact of the internal gut microbiome and external skin microbiome on human aging and how to best assess these across human populations?
8. What would the human aging exposome convey toward understanding of human health (both, wellbeing and disease) such as metabolic syndromes, cancer, cardiovascular, and neurodegenerative diseases?
9. How does the aging exposome research reach the stakeholders, governance, policy makers, health practitioners, and general population to realize the goals of this as global translational research endeavor?
10. Are we at a stage to sample personalized exposome or focus on community level stressors such as population, time, space, and region specific exposomes wrt. to aging research?
11. How would the findings from human aging exposome be used to change the human environment to help slower the process of biological or healthy aging?
12. Does the exposome affect males and females differently in humans and animal models, i.e., sex-specificity of the exposome?
13. Does the exposome of aging promises to deliver exposomal biomarkers of aging, independent of endogenous metabolism and genetics?
14. How can one leverage the exposomal accelerators of aging for identifying therapeutic targets in the scale of an individual (precision medicine) and at the population scale?
15. Does specific human genotypes and phenotypes predispose individuals to exposome- mediated aging to varied degrees?

Undoubtedly, exposomics allows one to study interactions between chronic stress and environmental chemicals that disrupt stress response pathways (i.e., “stressogens”) (Smith et al., 2015). In the future, multiple human cohorts and animal model based aging studies can be revisited form a systems biology stand point to capture the entirety of exposome and associate it with human aging. Clearly, in the future to capture the holistic exposome, one needs to obtain data from questionnaires (i.e., occupation, smoking history, habits, diseases, dietary choices), GIS-based environmental models (i.e., air pollution, heavy metals), images/pictures (i.e., usage of PHPs, food, cleaning products), mobile devices (i.e., smart phones to environmental sensors, wearable devices) among others to be able to obtain biomarkers across multiple time points and tissues following diverse approaches (Siroux et al., 2016). Approaches such as targeted and non-targeted exposomics efforts that look into dose-dependency and time-specific response to the exposome will be meaningful to understand the fate of a given chemical exposure, its transformation and degradation products during the course of human aging. Its also true that in spite of the availability of evidence on the role of chemical exposures affecting the process of aging, the precise cellular mechanisms and players such as proteins, lipids, nucleotides and carbohydrates as interacting partners of the exposome that negatively affect metabolism at an omics scale are largely understudied. Further, technologies and methods to capture these *in vivo* interactions are far from established.

## Concluding Remarks

In summary, one can safely claim that the vast majority of the studies in the past with “exposure” and “aging” have studied one exposure at a time against a specific aging type, i.e., effect of PM_2.5_ on skin aging or BPA exposure in rats. The number of studies that have looked at exposures in aging animals, and human population to decipher the association of exposures with aging are very limited. Top most exposures that are studied include air pollution (PM_2.5_), heavy metals, EDCs, organometallics, addiction compounds (alcohol, nicotine, cocaine), and drugs/medications, thus indicating that there is a myriad of unexplored opportunities in investigating the under studied exposures in relationship to human aging. However, the challenge would be to capture the near-entirety of the human chemical exposome via multiple analytical platforms and data-centric approaches that can complement the enormous challenges associated with capturing the human exposome. We are definitely on right track with high resolution and high throughput analytical platforms from mass-spectrometry and various spectroscopy methods to capture the chemical exposome, and a myriad of annotation tools that with newer *in silico* approaches and tools lend themselves for identification of new exposomal signatures. The past studies have taken a reductionist approach that may help only understand the role of a single variable, but those will inadequately capture the complexity of the exposome and its interactions. However, to capture such enormity of exposome, one needs multiple longitudinal studies, over ethnically diverse cohorts and experimental model systems for validation with large statistical power. There is a clear need for studying aging in not only entire systems level of an organism, but also for each organ and cell-type in the longer run for obtaining mechanistic insights. Connecting the exposome with aging, in a real word scenario where an individual is subjected to multiple exposures at any point of time, is challenging (i.e., for decomposing the effect of single exposures on aging) yet not impossible (i.e., with evidence and insights from animal model studies). Gained insights from animal model studies, cell cultures, and epidemiological studies need to be contextualized for realizing the goals of associating the chemical exposome with human aging in our engineered environment. Resolving the true “unknown unknowns” [i.e., defined as truly novel compounds that are not represented in spectral DBs and would need the use of computer-aided structure elucidation methods (Wishart, 2009)] from the captured exposome that associate with human aging is going to be critical in understanding the effect of modern human lifestyle on accelerated aging.

## Author Contributions

BBM conceived the idea, prepared, and wrote the original review.

## Conflict of Interest

The author declares that the research was conducted in the absence of any commercial or financial relationships that could be construed as a potential conflict of interest.

## Footnotes

1 https://www.norman-network.com/nds/SLE/

## Abbreviations

CE-MS: capillary electrophoresis mass spectrometry
DB: database
EPA: U.S. Environmental Protection Agency
GC-MS: gas chromatography-mass spectrometry
HMDB: Human Metabolome Database
HRMS: high resolution mass spectrometry
LC-MS: liquid chromatography mass spectrometry
MS: mass spectrometry
MS/MS: tandem mass spectrometry
NMR: nuclear magnetic resonance spectroscopy
VOC: volatile organic carbon.
